# wFLFM: enhancing the resolution of Fourier light-field microscopy using a hybrid wide-field image

**DOI:** 10.35848/1882-0786/abd3b7

**Published:** 2021-01-07

**Authors:** Wenhao Liu, Shu Jia

**Affiliations:** Wallace H. Coulter Department of Biomedical Engineering, Georgia Institute of Technology and Emory University, Atlanta, GA 30332, United States of America

## Abstract

We introduce wFLFM, an approach that enhances the resolution of Fourier light-field microscopy (FLFM) through a hybrid wide-field image. The system exploits the intrinsic compatibility of image formation between the on-axis FLFM elemental image and the wide-field image, allowing for minimal instrumental and computational complexity. The numerical and experimental results of wFLFM present a two- to three-fold improvement in the lateral resolution without compromising the 3D imaging capability in comparison with conventional FLFM.

Light-field microscopy (LFM) has rapidly emerged as a promising volumetric, high-speed imaging method.^1,2)^ Implementing a microlens array (MLA) on a conventional wide-field microscope, LFM can simultaneously capture the 2D spatial and 2D angular information of the incident light in a single camera frame, from which the entire volume of an object can be computationally reconstructed.^1,2)^ This imaging scheme effectively decouples the volume acquisition time from the spatial dimensions, making LFM a highly scalable approach to interrogating biological systems across different spatial levels, ranging from functional brain imaging^3–6)^ to single-cell studies.^7)^

Recently, growing interests have been focused on the development of Fourier light-field microscopy (FLFM), which can record the spatial and angular information in the Fourier (or the aperture) domain, thus allowing for spatially-invariant sampling and image formation.^8–11)^ The strategy effectively overcomes the fundamental limitations of LFM in reconstruction artifacts and computational cost, significantly enhancing the imaging capability and enabling versatile FLFM implementations.^12–14)^

However, FLFM remains restricted from broader applications by its intrinsic limitation as an aperture-partitioning system. In particular, the lateral resolution of a FLFM system can be estimated as $R_{xy}=\frac{\lambda N}{2\text{NA}}$, where *λ* is the wavelength of the emission light, NA is the numerical aperture of the objective, and *N* is the partitioning ratio between the aperture size and the diameter of a microlens.^11,15)^ In practice, *N* is larger than one to obtain the angular information (or perspective views) through multiple microlenses, which as a result, inevitably compromises the spatial resolution of FLFM compared with the diffraction limit (~*λ*/2NA) of its wide-field counterpart. In fact, several approaches have been reported to improve the resolution of light-field imaging combining a standard high-resolution image.^16–18)^ However, these developments rely primarily on conventional light-field implementations, and a strategy for FLFM remains unexplored.

Here, we introduce wFLFM, an approach that enhances the FLFM resolution using a hybrid wide-field image. Specifically, as FLFM forms elemental images through each partitioned segmentation of the aperture, the on-axis elemental image contains a consistent orthographic view while at a lower resolution with respect to conventional wide-field microscopy. In this sense, we reason that by replacing this on-axis component with the corresponding high-resolution wide-field image, the hybrid elemental images are able to provide enhanced spatial resolving power to FLFM without compromising the capture of angular information and thus the overall volumetric imaging capability.

As illustrated in Fig. 1(a), we constructed the wFLFM system on an epi-illumination wide-field microscope (Nikon Eclipse Ti-U) implemented with a 40×, 0.95NA objective lens (Nikon CFI Plan Apo Lambda 40XC) and a 647 nm laser (MPB). The fluorescence emission was collected through a dichroic mirror (T660lpxr, Chroma) and an emission filter (ET700/75, Chroma) and imaged separately from the two portals of the microscope in both wide-field and light-field paths. In the wide-field path, the native image plane (NIP) was recorded using an sCMOS camera (Hamamatsu ORCA-Flash4.0, pixel size *P*_cam_ = 6.5 *μ*m). In the light-field path, the NIP was then Fourier transformed in conjugation to the aperture plane of the objective lens using a Fourier lens (*f*_FL_ = 100 mm). The back focal plane of the Fourier lens was partitioned by an MLA (MLA-S600-f28, RPC Photonics; *f*_ML_ = 16.8 mm, NA_ML_ = 0.018), forming an array of elemental images on its back focal plane, which was recorded by an sCMOS camera (Andor Zyla 4.2, pixel size *P*_cam_ = 6.5 *μ*m).

As seen in FLFM, unlike conventional microscopy, the axial depths can be acquired as the variations in the composite spatial frequencies at the Fourier plane of the objective, resulting in lateral displacements of the corresponding images in a radially symmetric manner (except for the on-axis elemental image) [Fig. 1(b)].^10,11)^ Notably, as the aperture is partitioned, the effective NA in the formation of each elemental image is reduced from the objective lens. Using the theoretical model in Ref. 11, the lateral resolution of the FLFM system can be derived as $R_{xy}=\frac{\lambda }{{\text{2NA}}_{\text{FLFM}}}=\frac{\lambda }{{\text{2NA}}_{\text{ML}}}\times \frac{f_{\text{FL}}}{f_{\text{ML}}}\times \frac{1}{M}=2.81\;\mu \text{m}$, where the emission wavelength is 680 nm and the wide-field magnification *M* = 40. In addition, the sampling of the FLFM system is also compromised as the magnification $M_{\text{FLFM}}=M\frac{f_{\text{ML}}}{f_{\text{FL}}}=6.72$, corresponding to an effective pixel size as $P_{\text{FLFM}}=\frac{P_{\text{cam}}}{M_{\text{FLFM}}}=967\;\text{nm}$ (versus 162.5 nm as in the wide-field system). As a result, these factors lead to an on-axis FLFM elemental image of a lower resolution and sampling, in comparison with the same orthographic view taken at the diffraction limit using the wide-field path [Figs. 1(c) and 1(d)].

To overcome the limitation, we took advantage of the wide-field image and implemented the wFLFM approach in four main steps. First, we interpolated the FLFM elemental images to match the effective pixel size (*P*_cam_ /*M* = 162.5 nm) of the wide-field image. The corresponding simulated FLFM PSF at the same sampling rate can be generated for deconvolution to obtain a high-sampling reconstruction (termed HS-FLFM). Second, the on-axis elemental image was replaced by a wide-field image of the same field of view that contains in-focus projection of the same axial range with respect to FLFM. Here, we noticed that a better performance can be achieved using a wide-field image after deconvolving the original diffraction-limited wide-field image with its corresponding PSF. Third, the four nearest-neighboring elemental images of the on-axis image were removed to enhance the high spatial frequency components in the reconstruction. The intensity of the remaining elemental images was then scaled down in accordance with the new on-axis component to maintain a feasible weighting in the deconvolution process. Lastly, a hybrid 3D PSF was generated by replacing each axial layer of the on-axis component of the upsampled FLFM PSF by the image of the in-focus wide-field PSF. The intensity of the wide-field PSF was accordingly scaled on each layer to exhibit the same peak intensity of the corresponding FLFM PSF. With these procedures, the volume of the object can be retrieved using the FLFM reconstruction algorithm based on the Richardson–Lucy iteration scheme.^11)^

To validate wFLFM, we first numerically created a set of objects consisting of 9 rings with the same thickness of 1 pixel and the hollow centers ranging from 2 to 18 pixels at an increasing step of 2 pixels (Fig. 2). Using a pixel size of 162.5 nm, these rings exhibit inner diameters varying between 325 nm and 2.93 *μ*m, i.e. from slightly above the diffraction limit of wide-field microscopy (~358 nm) to above the resolution of FLFM (2.81 *μ*m) [Fig. 2(a)]. The wide-field image was created by initially convolving the ground truth with the wide-field PSF, which result was then deconvolved with the same PSF [Figs. 2(b) and 2(c)]. Next, the light-field images of each object were produced by convolving the ground truth with the PSF of FLFM and then resized to match the sampling of the wide-field image (e.g. Figures 2(d) and 2(e) for the largest ring). The hybrid images were generated by replacing the on-axis elemental image with the deconvolved wide-field image, removing its four nearest-neighboring elemental images, and scaling down 4× the intensity level of the four remaining elemental images to facilitate an optimum reconstruction quality [Fig. 2(f)].

The reconstructed results of FLFM, HS-FLFM, and wFLFM were obtained using their corresponding PSFs [Figs. 2(g)–2(i)]. It should be noted that conventional FLFM, as seen, is able to resolve the hollow structure of the ring at 2.76 *μ*m in diameter, agreeing with the predicted FLFM resolution of 2.81 *μ*m [Fig. 2(g)]. Meanwhile, HS-FLFM exhibits an enhanced resolution of the ring at 2.44 *μ*m in diameter, showing a readily noticeable improvement by solely recovering a better sampling rate of the light-field images [Fig. 2(h)]. In contrast, wFLFM exhibits a clear resolution of the ring at 812.5 nm in diameter, >3× improvement over the resolution of conventional FLFM [Fig. 2(i)].

Next, we demonstrated wFLFM experimentally using phantom samples. We placed 1 *μ*m dark-red fluorescent beads on the focal plane and recorded and processed their wide-field and light-field images [Figs. 3(a)–3(e)]. Here, prior to the same procedures as in the numerical demonstration, a deconvolved wide-field image was obtained and registered with the on-axis elemental image of HS-FLFM to correct any misalignment between the two portals [Figs. 3(b) and 3(c)]. As such, the on-axis elemental image in HS-FLFM [Fig. 3(e)] was substituted by the deconvolved wide-field image [Fig. 3(b)], and the hybrid images of wFLFM was generated by removing the four nearest-neighboring elemental images and scaling in intensity of the four remaining elemental images for optimum reconstruction [Fig. 3(f)]. The reconstruction results of FLFM, HS-FLFM and wFLFM were obtained using their corresponding numerical PSFs [Figs. 3(g)–3(i)]. Notably, for the two pairs of nearby beads separated by 2.51 *μ*m and 2.77 *μ*m [Fig. 3(a)], conventional FLFM can barely resolved the former pair as their separation is close to the FLFM resolution of 2.81 *μ*m [Fig. 3(g)]. Increasing the sampling rate (i.e. reducing the pixel size from 967 nm to 162.5 nm), HS-FLFM mitigated pixelated depiction of the 1 *μ*m objects with a moderately enhanced resolution of both pairs [Fig. 3(h)]. In contrast, wFLFM offers a clear visualization of the structures, in consistence with the wide-field image [Fig. 3(i)].

Lastly, we validated wFLFM using 250 nm dark-red fluorescent beads distributed at two axial depths on an attached glass slide and cover slide, as well as a 6 *μ*m surface-stained dark-red fluorescent microsphere. Here, to verify the usability of the approach on a high-resolution FLFM system, we replaced a 100×, 1.45NA objective lens (Nikon CFI Plan Apo Lambda 100×) and a Fourier lens mbi; [(*f*_FL_ = 75 mm)], which lead to a lateral FLFM resolution of 843 nm. To generate the wide-field image that contains in-focus volumetric projection of the same axial range, we rescaled the intensity of the wide-field focal stack with respect to the corresponding FLFM PSF on each layer, overlaid these wide-field images, and utilized the same procedures as above described to form the hybrid elemental images for reconstruction.

With the 250 nm beads, we retrieved the volume of the sample using both FLFM and wFLFM [Figs. 4(a) and 4(b)]. The measurements in conventional FLFM exhibited 3D FWHM values of individual beads at 569 ± 29 nm (*x*), 548 ± 48 nm (*y*) and 498 ± 9 nm (*z*) at *z* = −0.49 *μ*m, and 838 ± 30 nm (*x*), 617 ± 15 nm (*y*) and 1070 ± 47 nm (*z*) at *z* = 2.27 *μ*m. In contrast, the wFLFM measurements showed the corresponding 3D FWHM values of the same objects at 209 ± 28 nm (*x*), 226 ± 26 nm (*y*) and 448 ± 28 nm (*z*) at *z* = −0.49 *μ*m, and 254 ± 22 nm (*x*), 250 ± 12 nm (*y*) and 915 ± 106 nm (*z*) at *z* = 2.27 *μ*m. These results, in comparison with conventional FLFM, demonstrated a >2× enhancement in the lateral FWHM values with consistent axial measurements, allowing for an adequate resolution of two nearby beads separated as close as 768 nm located at *z* = 2.27 *μ*m using wFLFM [Figs. 4(c)–4(i)]. With the 6 *μ*m surface-stained microsphere, the reconstructed images using wFLFM exhibited the hollow spherical structure with a finer surface thickness, in comparison with the results using conventional FLFM [Figs. 4(j)–4(o)]. Measurements of the surface profiles showed the thickness reconstructed by wFLFM at 300–400 nm, a ~2× improvement over the 800–900 nm thickness obtained in the FLFM images. Notably, the reconstructed microsphere exhibited an elongated rugby-like axial pattern, which is consistent with previously reported numerical and experimental FLFM observation using a 40×, 0.95NA objective lens.^11)^ Finally, it should be mentioned that the wFLFM approach presented such an improvement at no expense in the axial information, mainly because of the fact that only the on-axis elemental image is substituted while the rest of the elemental images that consist of higher spatial frequencies (thus high angular sensitivity) are well maintained in the reconstruction process.

In summary, we have developed wFLFM to provide a 2–3× lateral resolution enhancement for FLFM by combining wide-field images. The principle of wFLFM exploits the nature of the FLFM image formation at the aperture plane, making it readily compatible with many commonly used wide-field modules and thus inducing minimum instrumental and algorithmic complexity. In particular, alternative to the use of focal stacks to form the in-focus wide-field image of the volume, the development of various extended depth of focus methods readily permits feasible acquisitions of such a high-resolution wide-field image in a scanningless or inertia-free manner across the DOF of FLFM for reconstruction of volumetric objects.^19)^ Exploring aperture-partitioning features, further developments are anticipated to achieve resolution enhancement in all three-dimensions, thereby advancing wFLFM as a particularly promising approach for interrogating biology with exquisite spatiotemporal resolving power and 3D capability, as well as at a high scalability, spanning broad molecular, cellular, and tissue systems.

## Figures and Tables

**Fig. 1.**
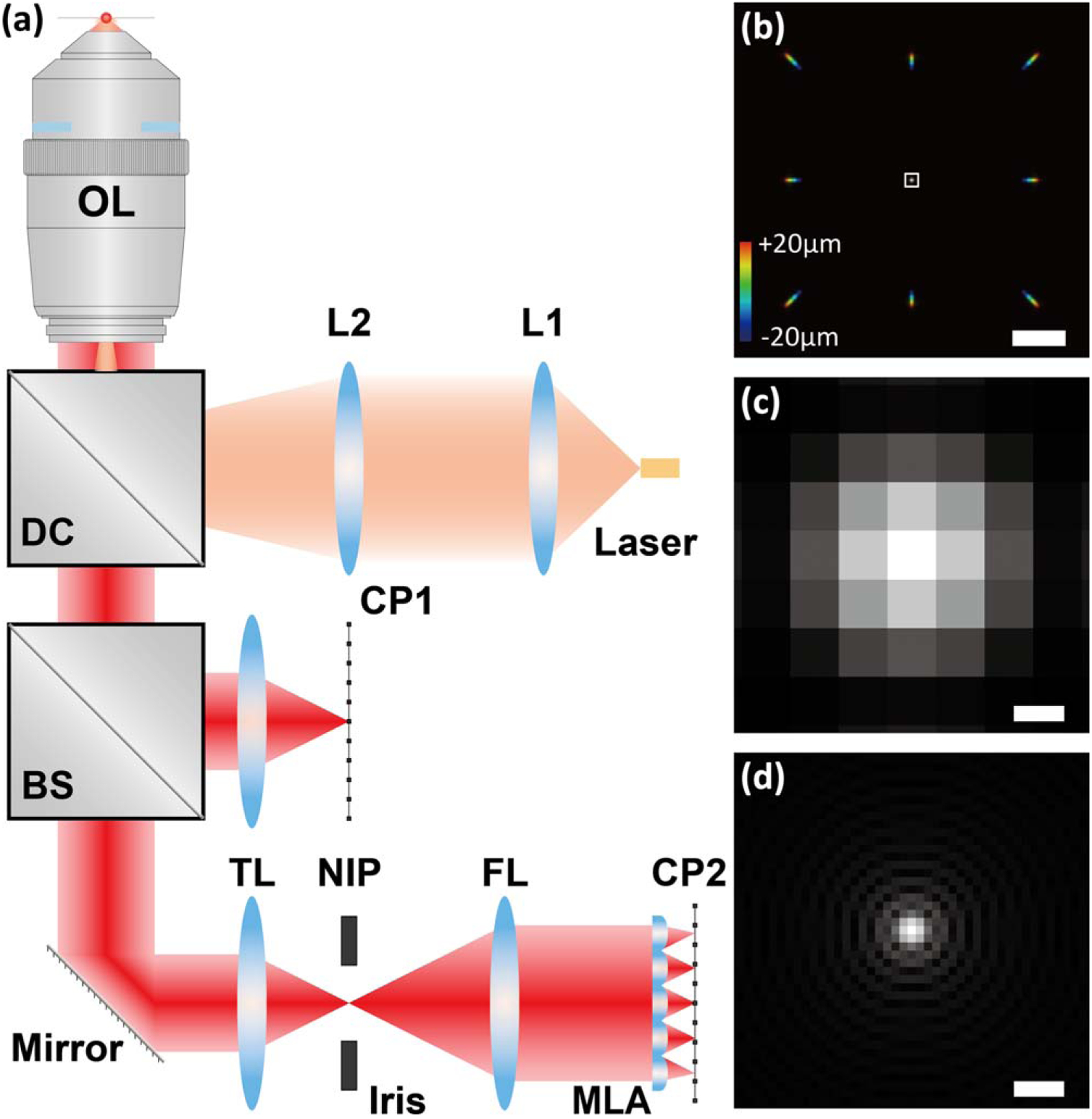
(a) A schematic of the wFLFM setup. The objective lens (OL) and the tube lenses (TLs) form images at the native image planes (NIPs) of both the wide-field and light-field paths. The light-field NIP area is adjusted by an iris and transformed by a Fourier lens (FL) to its back focal plane, where the microlens array (MLA) is situated. Both images are recorded by sCMOS cameras. DC, dichroic cube; BS, beam splitter, CP, camera plane. The inset diagram illustrates image formation through the MLA for emitters at different axial positions, implying recording of both the spatial and angular information in an uncompromised manner. (b) Axial stack projection (step size = 500 nm) of the simulated point-spread function (PSF) through a 3 × 3 MLA (effective pitch = 89.3 *μ*m in the object space) within an axial range from −20 to 20 *μ*m, as color-coded in the color scale bar. (c), (d) Zoomed-in image (c) of the boxed region in (b) (i.e. the on-axis elemental image at the focal plane) and the corresponding image (d) taken by the wide-field path. Scale bars: 40 *μ*m (b), 1 *μ*m (c), (d).

**Fig. 2.**
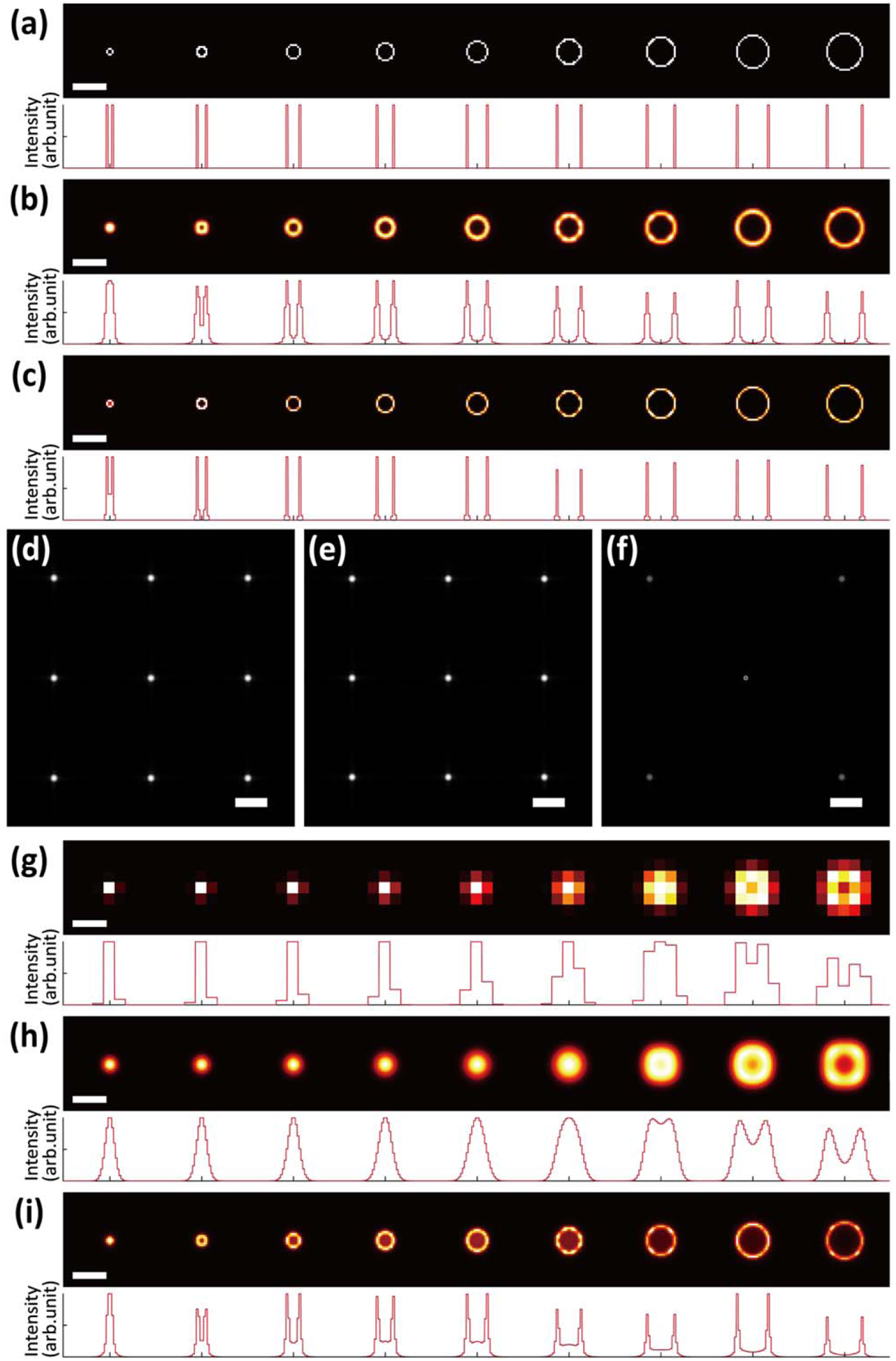
(Color online) (a)–(c) Top, numerical simulations of the ground-truth (a), wide-field (b), and deconvolved wide-field (c) images. Bottom, the corresponding cross-sectional profiles across the centers of each image. (d)–(f) Numerical simulations of the elemental images of conventional FLFM (d), HS-FLFM (e) and wFLFM (f) for the ring object at 3.1 *μ*m in diameter. (g)–(i) Top, reconstructed results of conventional FLFM (g), HS-FLFM (h) and wFLFM (i). Bottom, the corresponding cross-sectional profiles across the centers of each image. The results exhibited the best resolution achieved in the ring objects of 2.76 *μ*m (second largest), 2.44 *μ*m (third largest) and 812.5 nm (second smallest) in diameter in (g)–(i), respectively. Deconvolution of 30 iterations was used in all reconstructions. Scale bars: 3 *μ*m (a)–(c), (g)–(i), 200 *μ*m (d)–(f).

**Fig. 3.**
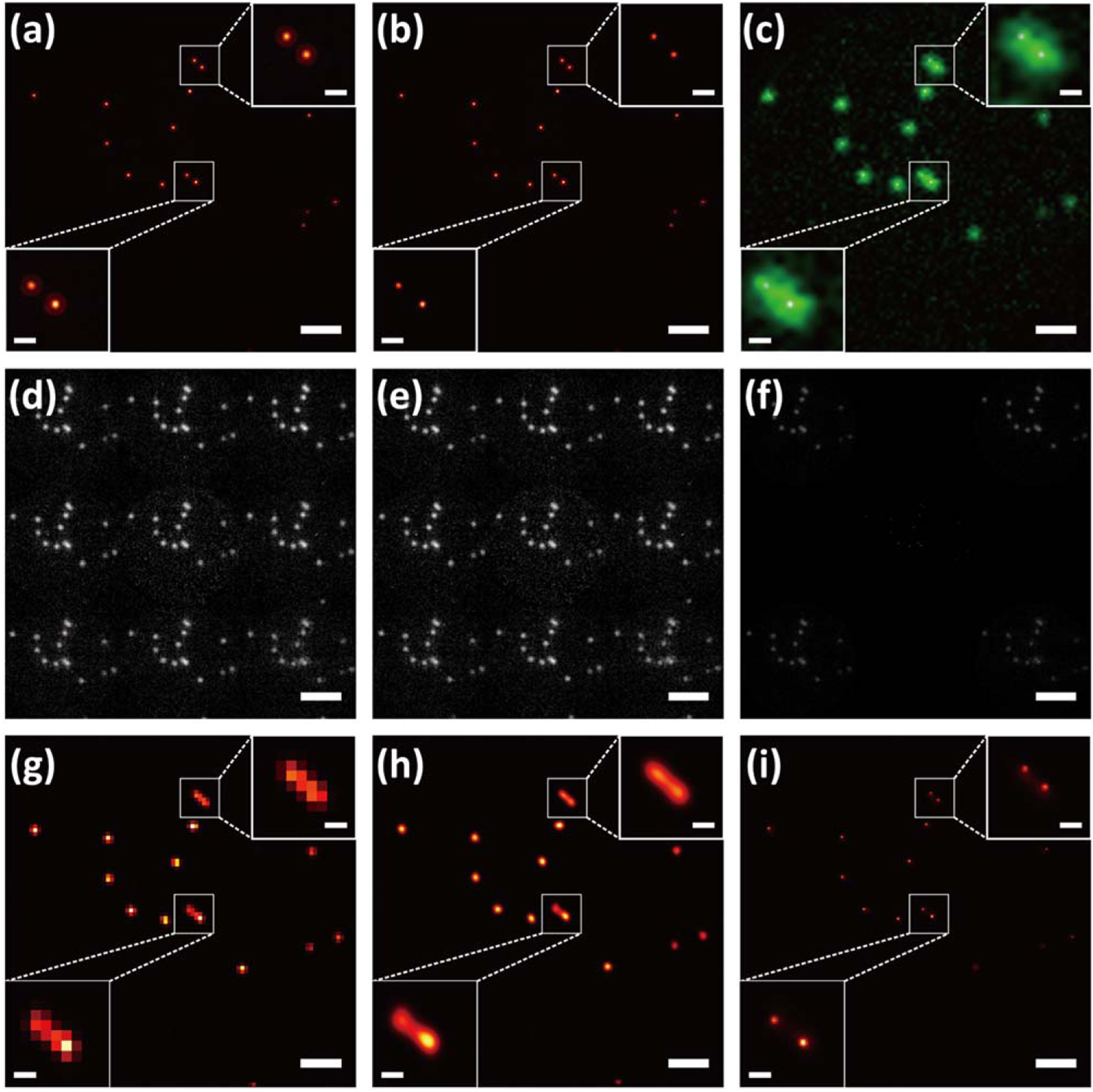
(Color online) (a), (b) Wide-field (a) and deconvolved wide-field (b) images of 1 *μ*m dark-red fluorescent beads. The inset images show two pairs of beads separated by 2.51 *μ*m (top right) and 2.77 *μ*m (bottom left). (c) Registration of (b) and the on-axis elemental image of HS-FLFM. (d)–(f) Elemental images of conventional FLFM (d), HS-FLFM (e) and wFLFM (f) of 1 *μ*m dark-red fluorescent beads. (g)–(i) Reconstructed images of (d)–(f), respectively. The inset images show the resolution of the two pairs of beads in each approach. Deconvolution of 30 iterations was used in all reconstructions. Scale bars: 10 *μ*m (a)–(c), (g)–(i), 2 *μ*m (insets in (a)–(c), (g)–(i), 200 *μ*m (d)–(f).

**Fig. 4.**
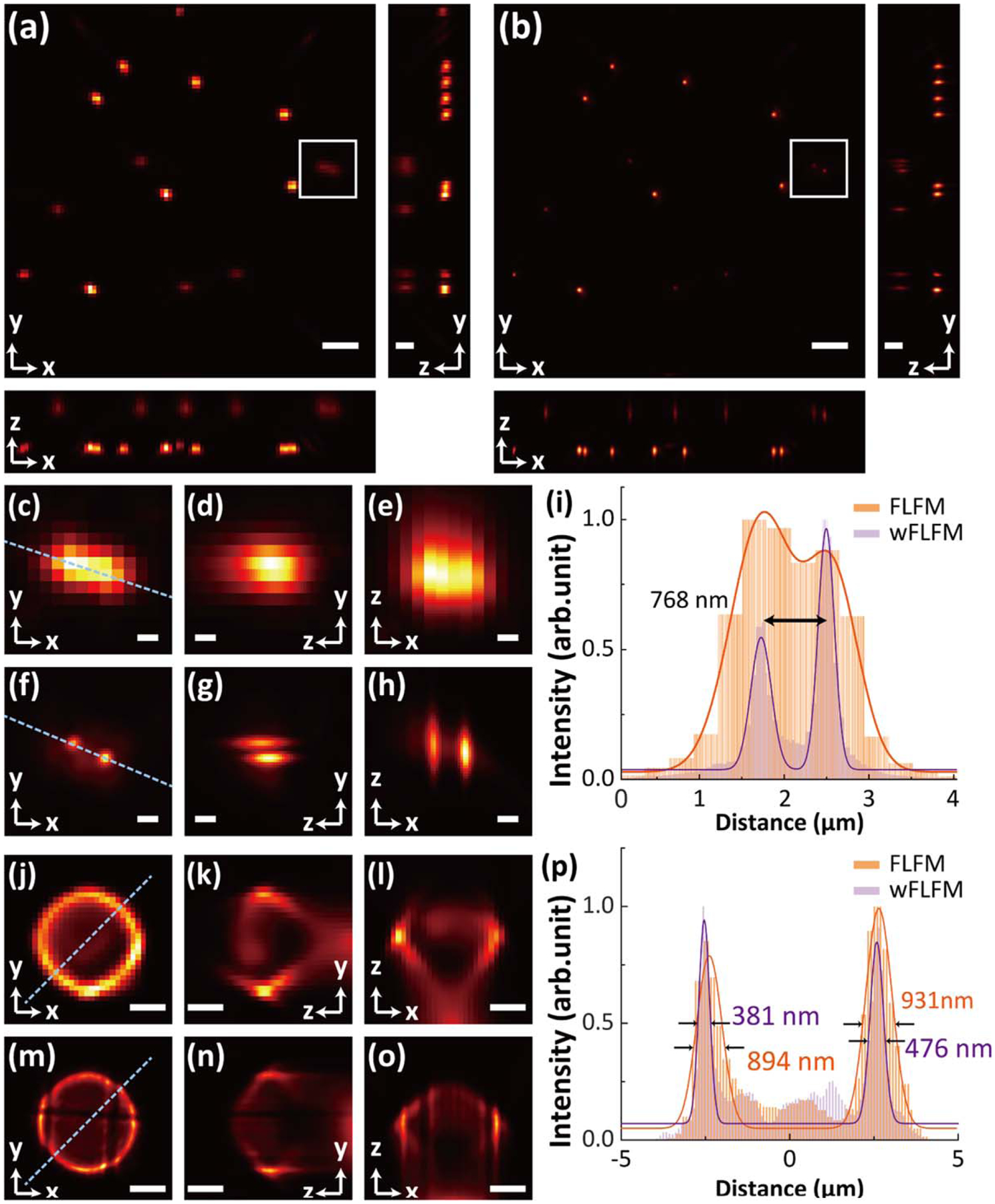
(Color online) (a), (b) Maximum-intensity projection (MIP) *x*–*y* images and the corresponding inset *x*–*z*, *y*–*z* views of the reconstructed volumes using conventional FLFM (a) and wFLFM (b). (c)–(e) Zoomed-in images in *x*–*y* (c), *y*–*z* (d), and *x*–*z* (e) of the boxed region in (a). (f)–(h) Zoomed-in images in *x*–*y* (f), *y*–*z* (g), and *x*–*z* (h) of the boxed region in (b). (i) Cross-sectional profiles along the dashed lines in (c) and (f), showing a clear resolution of two nearby beads separated by 768 nm using wFLFM. (j)–(o) Cross-sectional images of the reconstructed 6 *μ*m surface-stained microsphere using conventional FLFM (j)–(l) and wFLFM (m)–(o) in *x*–*y* (j), (m), *y*–*z* (k), (n), and *x*–*z* (l), (o). (p) Cross-sectional profiles along the dashed lines in (j) and (m), showing a 2× enhancement in the surface thickness using wFLFM. Deconvolution of 20 iterations was used in (a)–(i) and 30 iterations in (j)–(p). Scale bars: 2.6 *μ*m (a), (b), 1.3 *μ*m (insets in (a), (b), 500 nm (c)–(h), 2 *μ*m (j)–(o).
